# Integrating QTL mapping with transcriptome analysis mined candidate genes of growth stages in castor (*Ricinus communis* L.)

**DOI:** 10.1186/s12864-025-11348-9

**Published:** 2025-02-22

**Authors:** Guanrong Huang, Jiannong Lu, Xuegui Yin, Liuqin Zhang, Haihong Lin, Xiaoxiao Zhang, Chaoyu Liu, Jinying Zuo

**Affiliations:** College of Coastal Agricultural Sciences, Guangdong Ocean University, Zhanjiang, 524088 China

**Keywords:** Castor (*Ricinus communis* L.), Growth stages, QTL analysis, Differential expression analysis, WGCNA

## Abstract

**Background:**

The growth stages largely determine the crop yield, while little is known about their genetic mechanisms in castor. In this study, the QTL mapping and candidate gene mining of growth stages were conducted using populations F_2_ and BC_1_, combining with differential expression analysis and weighted gene co-expression network analysis (WGCNA). The traits studied included the emergence date (ED), the budding date of primary spike (PSBD), the flowering date of primary spike (PSFD), the maturation date of primary spike (PSMD), and the maturation date of primary branch spike (PBSMD).

**Results:**

A total of 20 QTLs conferring four traits (ED, PSBD, PSFD and PBSMD) were identified in the F_2_ population, with a phenotypic variation explained (PVE) of single QTL ranged from 0.24 to 25.46%. Five QTLs underlying PSMD and PBSMD were identified in the BC_1_ population, with a PVE of single QTL ranged from 4.74 to 10.82%. To our surprise, almost all the identified QTLs were clustered within two marker intervals, the RCM1769-RCM1838 on linkage group 6 and RCM950-RCM917 on linkage group 3. Subsequently, 473 open reading frames (ORFs) were searched out within these two clusters and 110 differentially expressed genes (DEGs) were screened out from these ORFs by the comparative transcriptome clean data (a total of 140.86 G) at the budding date, the initial flowering date and the full flowering date between parental racemes. With these DEGs, five distinct gene co-expression modules were generated using WGCNA. Showing significant differential expression between parents, four candidate genes (*LOC8261128*, *LOC8278994*, *LOC8281165* and *LOC8259049*) in module MEturquoise, were recognized and were annotated as *RcSYN3*, *RcNTT*, *RcGG3* and *RcSAUR76* respectively. This finding implies their potential role in regulating the growth stages of castor.

**Conclusion:**

In this study, numerous QTLs conferring growth stages were detected and four candidate genes were mined, which need to be functionally validated. The results provided a new insight into the genetic structure of ED, PSBD, PSFD, PSMD and PBSMD, offered the candidate genes and molecular markers for their improvement as well in castor.

**Supplementary Information:**

The online version contains supplementary material available at 10.1186/s12864-025-11348-9.

## Introduction

Castor plant (*Ricinus communis* L.) is an important industrial oil crop with seed oil content accounting for 46 to 55%, which is widely grown in tropical, subtropical and temperate regions [1]. Ricinoleic acid, the most important component of castor oil (more than 85%), is a special hydroxyl fatty acid, which makes castor oil widely used in aerospace, machinery manufacturing, textile, printing, dyeing, medicine and other fields [2, 3]. With the rapid development of economy, the demand for castor oil is increasing steadily worldwide [2]. Castor oil is extracted by pressing castor seed, which is a commercially important source of castor oil [4, 5]. In the past decades, although some excellent achievements have been made, the genetic research and variety improvement of castor are still lagging behind on the whole [6], resulting in a lower planting benefit and a decreasing planting area year by year [7]. Therefore, breeding high-yielding varieties is of great significance to the development of castor industry.

Flowering date (FD) and maturation date (MD) largely determine castor yield, their enough cumulative days ensure that castor individuals have vigorous growth potential and harvest more plump seeds (i.e., decreased shriveled seed rate), which in turn increases overall yield [8, 9]. They have a potential role in reducing the cultivation cost and enhancing adaptability, especially FD, which is the key stage for the transition from vegetative growth to reproductive growth in flowering plants. In addition, seed filling date is also an important factor in determining castor yield, and seed filling duration significantly affects seed size and seed weight [10, 11]. Understanding the genetic structure of castor growth stages, with available functional gene resources, is conducive to the development of breeding strategy for purposeful cross-breeding; moreover, it is also available to accelerate the breeding of early-maturing and high-yielding castor varieties by means of technologies genome-modification, transgenic, molecular markers-assisted selection and multi-omics [6]. Through generation mean analysis, it was initially found that castor FD and MD were jointly controlled by major genes and polygenes, and conformed to the additive-dominant-epistatic inheritance model [9, 12–14]. The additive effect was predominant in castor FD, whereas the additive and non-additive effects were equal in castor MD [9, 12–14]. Furthermore, 183 possible flowering-related genes were tentatively found [15–17]. Nonetheless, few available gene resources (i.e., genes *RcNF-YB8*, *PLC2*, *PLC2M*, *PLC2N*, *PLC4*, *PLC4X2* and *PLC6*) can be applied in castor molecular breeding [16, 17].

FD and heading date (HD) have been extensively studied in *Arabidopsis* and rice respectively, which are susceptible to the environment (including photoperiodic, temperature, hormonal, stress and nutrient availability) [18–20]. Firstly, the photoperiodic pathway is the most important induction factor, which mediates HD through pathways *GIGANTEA*-*Heading date 1*-*Heading date 3a* (GI-Hd1-Hd3a) and *Grain number*,* plant height*,* and heading date 7*-*Early heading date 1*-*Hd3a/RICE FLOWERING LOCUS T 1* (Ghd7-Ehd1-Hd3a/RFT1) in rice, the former corresponding to the *GI*-*CONSTANS*-*FLOWERING LOCUS T* (GI-CO-FT) pathway that regulates FD in *Arabidopsis thaliana* [19]. In fact, many genes that have been recently identified regulate rice HD by promoting/repressing the expression of the above pathway members, such as *Early heading date 5* (*Ehd5*) [21], *Small Auxin Up RNA 56* (*SAUR56*) [22], *Flowering Locus T-like 12* (*FTL12*) [23], *Late Heading Date 3* (*LHD3*) [24]. Secondly, the temperature is an effective factor in predicting HD, e.g., *Pseudo-Response Regulator 37* (*PRR37*) represses flowering when the mean ambient temperatures fall below a critical threshold, while reverts to a flowering promoter at higher temperatures [25]. Thirdly, plant hormones, e.g., auxin [26], gibberellins [27], cytokinins [28], abscisic acid [29], brassinosteroids [30] and ethylene [31], have significant effects on plant flowering; Fourthly, abiotic stresses (including drought, salt and temperature) affect plant flowering mainly via the Ghd7-Ehd1-Hd3a/RFT1 pathway [18]. Fifthly, nutrients also affect plant flowering, a moderate amounts of potassium and phosphorus promote flowering while a low or high nitrogen delays flowering [32, 33]. Additionally, numerous genes have also been mined in other crops and even revealed crop-specific flowering pathways, e.g., genes, *TraesCS2A02G181200* [34], *constans of Zea mays1* (*conz1*) [35], *FANTASTIC FOUR gene family members* (*FAFs*) [36] and *Hd3a* [37], control flowering in wheat, maize, tomato and perilla respectively; modules BnTFL1-BnGF14nu-BnFD [38] and FvemiR160-FveARF18A-FveAP1/FveFUL [39] regulate flowering in rapeseed and woodland strawberry respectively.

In this study, mapping QTLs conferring emergence date (ED), budding date of primary spike (PSBD), flowering date of primary spike (PSFD), maturation date of primary spike (PSMD) and maturation date of primary branch spike (PBSMD) was performed in populations F_2_ and BC_1_ with methods composite interval mapping (CIM) and inclusive composite interval mapping (ICIM); And then, transcriptome sequencing on racemes of both parents at different stages was conducted for mining candidate genes within QTL clusters. It is expected to provide a reference for molecular marker-assisted selection and genetic function identification of growth stages in castor.

## Materials and methods

### Materials

Populations F_2_ and BC_1_ were constructed in a short cycle, with low cost and simple operation; moreover, the former had a large phenotypic segregation range and abundant genetic recombination, and the latter could be used to validate detected QTLs. Therefore, F_2_ and BC_1_ were selected as the mapping populations in this study. Two inbred lines, 9048 (P_1_, 25 individuals) and 16–201 (P_2_, 25 individuals), and three populations derived from them, i.e., F_1_ (25 individuals), F_2_ (282 individuals) and BC_1_ (F_1_ backcross with P_2_, 250 individuals), were used in this study. 9048, a pistillate line, was the female parent of Zibi 5, a main cultivar in China, with an earlier ED and a later budding date, flowering date and maturation date (Fig. S1); In contrast, 16–201 was a monecious line with a later ED, but with an earlier budding date, flowering date and maturation date than 9048. So, the BC_1_ population was constructed using 16–201 backcrossed to F_1_, in the hope that we could screen a group of materials within this population that significantly early flowering and other traits not much different from 9048. All the populations were planted at the experimental base of Guangdong Ocean University, Mazhang, Zhanjiang, Guangdong, China in September, 2020. Besides, populations P_1_ (15 individuals) and P_2_ (15 individuals) were planted again in November, 2023 for collecting transcriptome sequencing samples. The plant and row spacing was one meter. The cultivation management was same as high-yield field.

### Phenotype investigation

The days from sowing to castor cotyledon spreading, budding of primary spike, bloom of 50% female flowers on primary spike, maturation of 50% capsules on primary spike, and maturation of 50% capsules on primary branch spike were recorded as ED, PSBD, PSFD, PSMD and PBSMD respectively. Statistical description and Student’s *t* test were run by software SPSS 25 and Excel 2021.

### DNA extraction, genotyping and genetic map construction

Genomic DNA was extracted through a modified CTAB method, as suggested by Agyenim-Boateng et al. (2019) [40]. Five hundred sixty-six pairs of SSR (Simple sequence repeats) primers (Fig. S2), with clear and stable bands, were used in this study, which uniformly distributed on the whole castor genome and were selected from 1750 pairs of SSR primers developed based on the castor scaffolds JCVI_RCG_1.1 (BioProject: PRJNA16585) by the castor research group of Guangdong Ocean University [41]. Polymorphic primer screening and population genotyping were carried out according to the procedures described by Huang et al. (2023) [42] and Yeboah et al. (2021) [43] respectively.

The genetic map of F_2_ population was constructed using QTLIcimapping v4.2 software, using population model 7 to process population genotyping data and the Kosambi function to calculate the genetic distance, setting the LOD threshold value to eight. The genetic map of BC_1_ population was constructed similarly with the population model 2, and the linkage groups were determined according to the genetic map of F_2_ population and the marker information.

### QTL analysis

Under the condition of a better balance of false positives and false negatives and statistical significance, in order to detect more QTLs, single locus QTLs were mapped using the CIM method in WinQTLCart v2.5 software and the ICIM-ADD method in QTLIcimapping v4.2 software, with a LOD value of two [44]; Meanwhile, epistatic QTLs were identified using the ICIM-EPI method in QTLIcimapping v4.2 software with default parameters [45]. Confidence intervals for all QTLs were determined with 95% confidence. The QTLs with a phenotypic variation explained (PVE) more than 10% were defined as main-effect QTL. All detected QTLs were named according to the format described by Huang et al. (2023) [42], i.e., started with “q”, followed by trait abbreviation, chromosome serial number and QTL serial number on the chromosome; In addition, the capital “F” and “B” were prefixed the QTLs detected in populations F_2_ and BC_1_ respectively.

### RNA extraction and transcriptome sequencing

Racemes of 9048 and 16–201 were collected at the budding date (BD), the initial flowering date (IFD, 25% female flowers bloomed on the raceme) and the full flowering date (FFD, 75% female flowers bloomed on the raceme) respectively, with three biological replicates. Total RNA was extracted using RNAprep Pure Plant Plus Kit (Cat No. DP441, TIANGEN, Beijing, China) following the manufacturer’s protocol. In total, 18 RNA-seq libraries (two parents × three sampling stages × three biological replicates) were constructed and sequenced on the Illumina sequencing platform by Metware Biotechnology Co., Ltd. (Wuhan, China). Raw reads with adapter or N content over 10% or the number of low-quality bases more than 50% were filtered; And then, sequencing error rate and GC content distribution were conducted to generate clean reads. The clean reads were aligned to a reference castor genome ASM1957865v1 (BioProject: PRJNA589181) [46] by the software HISAT v2.2.1 with default parameters.

### Candidate gene prediction and expression analysis

Differentially expressed genes (DEGs) were screened in the two QTL clusters with |log2 Fold Change (FC)| ≥ 1 and false discovery rate (FDR) ≤ 0.05. Weighted gene co-expression network analysis (WGCNA) was performed on the Metware Cloud platform (https://cloud.metware.cn) using default parameters. Combining the genomic annotation information (including BlastP annotation and Swissprot annotation) and available literature description, candidate genes were expected to be found.

Relative expression levels of the selected candidate genes were assayed in racemes used for transcriptome sequencing. Except for the internal reference gene *Glyceraldehyde 3-phosphate dehydrogenase* (*GAPDH*) [43], all primers were designed by the online tool Primer-blast (https://www.ncbi.nlm.nih.gov/tools/primer-blast/) (Supplementary Table S1). cDNA was synthesized with one µg total RNA using the PrimeScript™ RT reagent kit (Cat No. RR047A, TAKARA, Japan). Quantitative real-time polymerase chain reaction (qRT-PCR) was carried out according to the procedures described by Yeboah et al. (2021) [43]. The internal reference gene was amplified in parallel with each candidate gene, repeated three times. The relative expression level of the candidate genes was calculated using the 2^−ΔΔCt^ method [47], and shown as mean and standard deviation.

## Results

### Phenotype analysis

**Fig. 1:**
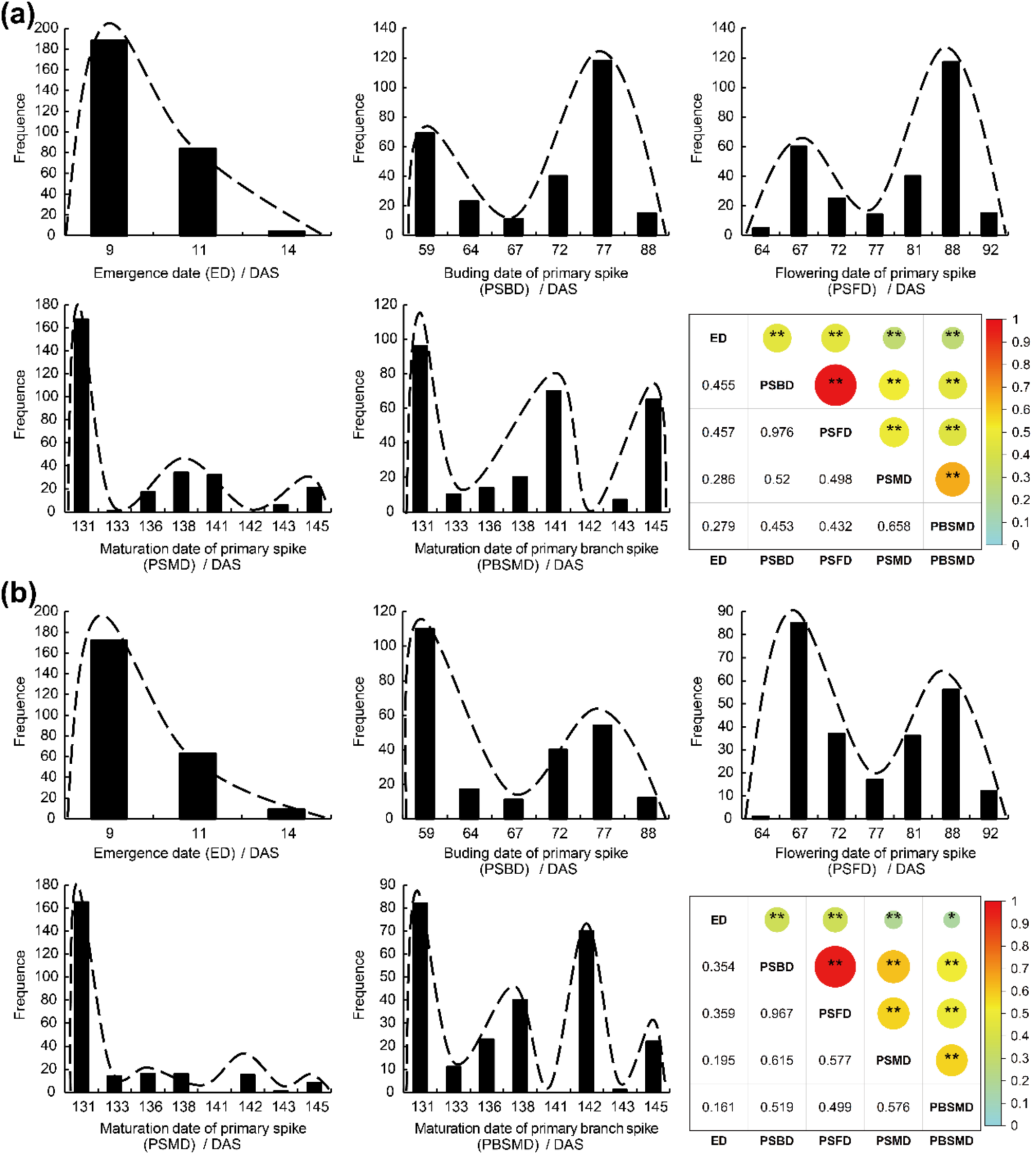
Frequency distribution and correlation analysis in populations F_2_ (**a**) and BC_1_ (**b**). For correlation analysis, reflecting the strength of the correlation by the size of the shaded area and are colored from red (coefficient = 1) to cyan (coefficient = -1); * and ** refer to significant and extremely significant correlation respectively

PSBD, PSFD, PSMD and PBSMD of 9048 were significantly later than that of 16–201 (*p* < 0.01), mostly due to the fact that the primary stem and branch stem of 9048 need to form more nodes than 16–201 to grow racemes (Table 1, Fig. S1). As a whole, these traits (i.e., ED, PSBD, PSFD, PSMD and PBSMD) showed a unidirectional transgressive inheritance. All traits displayed a multi-peaked continuous distribution or left skewed continuous distribution in populations F_2_ and BC_1_ (Fig. 1), implying the existence of major genes controlling ED, PSBD, PSFD, PSMD and PBSMD in castor [48]. There were significant positive correlations between these five traits in these two segregating populations (*p* < 0.01) (Fig. 1).

**Table 1:** Description statistics of growth stages

Trait	Parent			F_2_ population					BC_1_ population				
	9048 (*P*_1_)	16–201 (*P*_2_)	Difference	Range	Mean ± SD	CV (%)	Skewness	Kurtosis	Range	Mean ± SD	CV (%)	Skewness	Kurtosis
ED	9.18 ± 0.40	12.93 ± 0.47	-3.75 ^a^	9–14	9.68 ± 1.06	10.93	1.41	1.96	9–14	9.70 ± 1.21	12.50	1.85	3.40
PSBD	57.73 ± 2.41	53.14 ± 2.54	4.58 ^a^	59–88	70.89 ± 8.50	11.99	-0.13	-0.98	59–88	67.25 ± 8.81	13.10	0.60	-0.77
PSFD	76.64 ± 2.11	64.79 ± 1.63	11.85 ^a^	64–92	80.20 ± 9.11	11.36	-0.45	-1.41	64–92	76.56 ± 8.98	11.73	0.30	-1.48
PSMD	138.64 ± 1.43	120.50 ± 2.98	18.14 ^a^	131–145	134.64 ± 4.88	3.63	0.89	-0.71	131–145	133.17 ± 3.96	2.97	1.74	1.81
PBSMD	145.36 ± 0.81	133.79 ± 1.63	11.58 ^a^	131–145	137.82 ± 5.66	4.10	-0.09	-1.62	131–145	137.05 ± 5.07	3.70	0.03	-1.52

Student’s *t* test was done between 9048 and 16–201

*ED* emergence date, days after sowing (DAS), *PSBD* budding date of primary spike, DAS, *PSFD* flowering date of primary spike, DAS, *PSMD* maturation date of primary spike, DAS, *PBSMD* maturation date of primary branch spike, DAS

^a^ indicates significance level at 0.01

### Genetic map construction

The genetic maps of populations F_2_ and BC_1_ contained 63 and 33 SSR markers, with an average marker interval of 10.21 cM and 13.31 cM and a LOD value of eight and three, including ten and six linkage groups, covered 643.36 cM and 439.25 cM of the genome respectively (Fig. S3).

### QTL mapping with methods CIM and ICIM

**Fig. 2:**
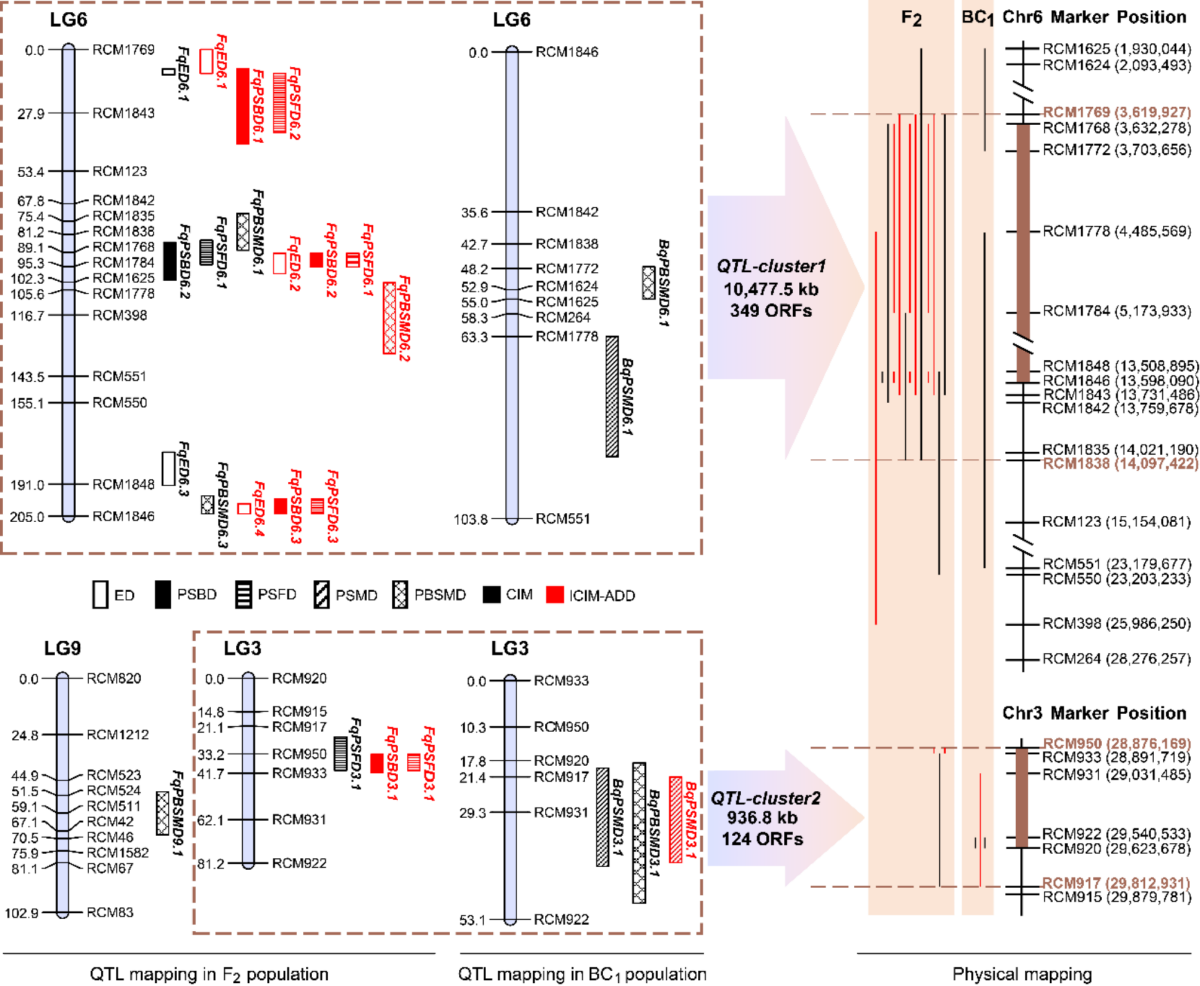
Distribution map of QTLs conferring growth stages in castor. For physical mapping, the black lines are the CIM mapping results and the red lines are that in ICIM

A total of eight and four QTLs were detected by the CIM method in populations F_2_ and BC_1_ respectively (Fig. 2; Table 2). They were distributed on linkage group 3, 6 and 9, with a PVE of single QTL ranged from 0.24 to 25.46%.

**Table 2:** QTLs detected in populations F_2_ and BC_1_

Pop	Trait	Method	QTL	LG	Pos.	Add.	Dom.	LOD	PVE (%)	CI	MI
F_2_	ED	CIM	*FqED6.1*	6	10.01	-0.12	-2.13	38.41	0.24	8.5–11.1	RCM1769-RCM1843
			*FqED6.3*	6	190.11	-0.72	0.43	3.62	25.46	176.9-191.5	RCM550-RCM1848
		ICIM	*FqED6.1*	6	2	-0.15	-0.46	2.39	4.06	0-10.5	RCM1769-RCM1843
			*FqED6.2*	6	92	0.15	-0.50	2.44	4.28	89.5–98.5	RCM1768-RCM1784
			*FqED6.4*	6	204	-0.05	0.53	2.86	4.16	199.5–204	RCM1848-RCM1846
	PSBD	CIM	*FqPSBD6.2*	6	89.21	-2.85	-1.32	3.36	1.85	84.7-101.3	RCM1838-RCM1625
		ICIM	*FqPSBD6.1*	6	18	-2.86	2.59	2.29	3.97	8.5–41.5	RCM1769-RCM1843
			*FqPSBD6.2*	6	90	-1.56	-3.85	5.52	4.72	89.5–95.5	RCM1768-RCM1784
			*FqPSBD6.3*	6	202	-0.24	6.55	7.42	7.81	197.5–204	RCM1848-RCM1846
			*FqPSBD3.1*	3	36	-1.35	4.05	3.25	3.58	33.5–41.5	RCM950-RCM933
	PSFD	CIM	*FqPSFD6.1*	6	89.21	-3.16	-1.35	3.53	2.22	83.8–94.5	RCM1838-RCM1784
			*FqPSFD3.1*	3	34.21	-1.33	3.28	2.10	4.34	25.9–40.5	RCM917-RCM933
		ICIM	*FqPSFD6.2*	6	17	-3.61	3.88	2.92	5.58	10.5–36.5	RCM1769-RCM1843
			*FqPSFD6.1*	6	91	-1.35	-4.07	4.63	3.75	89.5–95.5	RCM1768-RCM1784
			*FqPSFD6.3*	6	202	0.19	6.38	6.85	6.49	197.5–204	RCM1848-RCM1846
			*FqPSFD3.1*	3	36	-1.60	4.28	3.20	3.25	33.5–40.5	RCM950-RCM933
	PBSMD	CIM	*FqPBSMD6.1*	6	74.81	-1.95	-1.29	3.09	1.75	72.2–88.2	RCM1842-RCM1768
			*FqPBSMD6.3*	6	202.01	-1.41	3.94	2.95	6.36	196.1–204	RCM1848-RCM1846
			*FqPBSMD9.1*	9	59.11	-1.42	-0.46	2.15	2.14	49.9–68.8	RCM523-RCM42
		ICIM	*FqPBSMD6.2*	6	110	-1.30	-1.12	2.16	3.35	102.5-133.5	RCM1778-RCM398
BC_1_	PSMD	CIM	*BqPSMD6.1*	6	73.31	3.54	-	2.10	10.82	63.3–90	RCM1778-RCM551
			*BqPSMD3.1*	3	29.31	2.23	-	4.24	7.71	19.5–41.3	RCM920-RCM922
		ICIM	*BqPSMD3.1*	3	29	2.10	-	3.67	7.18	21.5–40.5	RCM917-RCM931
	PBSMD	CIM	*BqPBSMD6.1*	6	49.21	-3.61	-	2.26	4.56	47.8–55	RCM1772-RCM1625
			*BqPBSMD3.1*	3	33.31	2.25	-	2.14	4.74	18.3–49.5	RCM920-RCM922

Pop, LG, Pos., Add., Dom., CI and MI are abbreviations for population, linkage group, position, additive effect, dominance effect, confidence interval, and marker interval respectively

The trait description is the same as in Table 1

In F_2_ population, two, one, two and three QTLs underlying ED, PSBD, PSFD and PBSMD were identified, with the PVE of single QTL ranged from 0.24 to 25.46%, 1.85%, 2.22–4.34% and 1.75–6.36% respectively. No QTL conferring PSMD was detected. Numerous minor-effect QTLs and one main-effect QTL (*FqED6.3*) with a PVE of 25.46% were found.

In BC_1_ population, two QTLs conferring PSMD were detected with the PVE of single QTL ranged from 7.71 to10.82%; as well as two QTLs underlying PBSMD with the PVE of single QTL ranged from 4.56 to 4.74%. No QTL conferring ED, PSBD and PSFD was detected. Only one main-effect QTL (*BqPSMD6.1*) with a PVE of 10.82% was found.

A total of 12 and one QTLs were detected by the ICIM method in populations F_2_ and BC_1_ respectively (Fig. 2; Table 2). They were distributed on linkage group 3 and 6, with a PVE of single QTL ranged from 3.25 to 7.81%. Many QTLs were simultaneously detected by the CIM and ICIM-ADD methods.

In F_2_ population, three, four, four and one QTLs underlying ED, PSBD, PSFD and PBSMD were identified, with the PVE of single QTL ranged from 4.06 to 4.28%, 3.58–7.81%, 3.25–6.49% and 3.35% respectively. No QTL conferring PSMD was detected. Although no main-effect QTLs were found, most of the QTLs detected with PVE approached 4%, especially, four QTLs (with a total PVE of 20.08%) underlying PSBD and four QTLs (with a total PVE of 19.07%) conferring PSFD were found.

Only one QTL (*BqPSMD3.1*) conferring PSMD was identified in BC_1_ population, with a PVE of 7.18%.

### QTL clusters

All the detected QTLs were distributed on linkage group 3 and 6 except for *FqPBSMD9.1*, although there were other linkage group on the genetic map with higher density markers than these two linkage groups (Fig. S3). Notably, after mapping the above QTLs to the castor genome ASM1957865v1, they were found to be located within the marker interval RCM1769-RCM1838 (10,477.5 kb) on chromosome 6 and the marker interval RCM950-RCM917 (936.8 kb) on chromosome 3, named as *QTL-cluster1* and *QTL-cluster2* respectively (Fig. 2). Summarily, these two QTL clusters consisted of 18 and six allelic QTLs with the PVE of single QTL ranged from 0.24 to 25.46% and 3.25–7.71%, shared by five and four traits, containing 349 and 124 open reading frames (ORFs) respectively (Fig. 2; Table 3), which revealed the existence of the gene pleiotropy or close linkage between genes controlling ED, PSBD, PSFD, PSMD and PBSMD, and the genetic foundation of significant correlation among these five traits in castor (Fig. 1).

**Table 3:** Information of QTL clusters

QTL cluster	*QTL-cluster1*	*QTL-cluster2*
Population	F_2_ / BC_1_	F_2_ / BC_1_
Position	marker interval RCM1768-RCM1846 in chromosome 6	marker interval RCM950-RCM920 in chromosome 3
LOD	2.15–38.41 / 2.10–2.26	2.10–3.25 / 2.14–4.24
PVE (%)	0.24–25.46 / 4.56–10.82	3.25–4.34 / 4.74–7.71
Shared by	ED, PSBD, PSFD, PBSMD / PSMD, PBSMD	PSBD, PSFD / PSMD, PBSMD
Allelic QTL	*FqED6.1*, *FqED6.2*, *FqED6.3*, *FqED6.4*, *FqPSBD6.1*, *FqPSBD6.2*, *FqPSBD6.3*, *FqPSFD6.1*, *FqPSFD6.2*, *FqPSFD6.3*, *FqPBSMD6.1*, *FqPBSMD6.2*, *FqPBSMD6.3* / *BqPSMD6.1*, *BqPBSMD6.1*	*FqPSBD3.1*, *FqPSFD3.1* / *BqPSMD3.1*, *BqPBSMD3.1*
Number of allelic QTL with a PVE over 4%	9 / 2	1 / 3
Main-effect QTL	*FqED6.3* / *BqPSMD6.1*	-

The trait description is the same as in Table 1

### Epistatic QTL analysis

In F_2_ population, a total of 34 pairs of epistasis QTLs were identified (Supplementary Table S2). Among them, 25, one, three, four and one pairs of epistasis QTLs conferring ED, PSBD, PSFD, PSMD and PBSMD, with a PVE of each pair of QTLs ranged from 0.71 to 6.98%, 8.88%, 10.25–12.40%, 10.23–11.39% and 18.74% respectively. And four QTLs (*FqED6.1*, *FqED6.2*, *FqPSBD6.1* and *FqPSFD6.2*) possessed both epistatic and single-locus effects.

In BC_1_ population, a total of six pairs of epistasis QTLs were detected (Supplementary Table S3). Of which, two and four pairs of QTLs conferring ED and PSMD, with a PVE of each pair of QTLs ranged from 3.44 to 4.02% and 3.32–3.54% respectively. And two QTLs (*BqPSMD6.1* and *BqPSMD3.1*) possessed both epistatic and single-locus effects. No epistasis QTL underlying PSBD, PSFD and PBSMD was identified. With a percentage from 30.67 to 100% (Table 4), the epistasis effect was the important genetic component of castor ED, PSBD, PSFD, PSMD and PBSMD.

**Table 4:** Percentage of epistatic effect

Population	Trait	SE (%)	EE (%)	*P* (%)
F_2_	ED	12.50	69.49	84.76
	PSBD	20.07	8.88	30.67
	PSFD	19.07	34.88	64.66
	PSMD	-	43.59	100
	PBSMD	3.35	18.74	84.84
BC_1_	ED	-	7.46	100
	PSMD	7.18	13.87	65.88

*SE* PVE of single locus effect, *EE* PVE of epistasis effect, *P* percentage of epistatic effect

The trait description is the same as in Table 1

### Mining candidate genes combined with transcriptome analysis

In this study, 18 raceme RNA-seq libraries were constructed. After raw data filtering (i.e., filtering adaptor sequences and low base quality sequences), sequencing error rate checking (0.01%) and GC content distribution checking (the average GC content was 43.34%), a total of 140.86 G clean data were obtained by the Illumina sequencing, and the average Q20 and Q30 were 98.86% and 96.58% respectively (Supplementary Table S4). After the clean data were aligned to the castor genome ASM1957865v1, the total, mapped, unique-mapped and multi-mapped reads were shown (Supplementary Table S5).

**Fig. 3:**
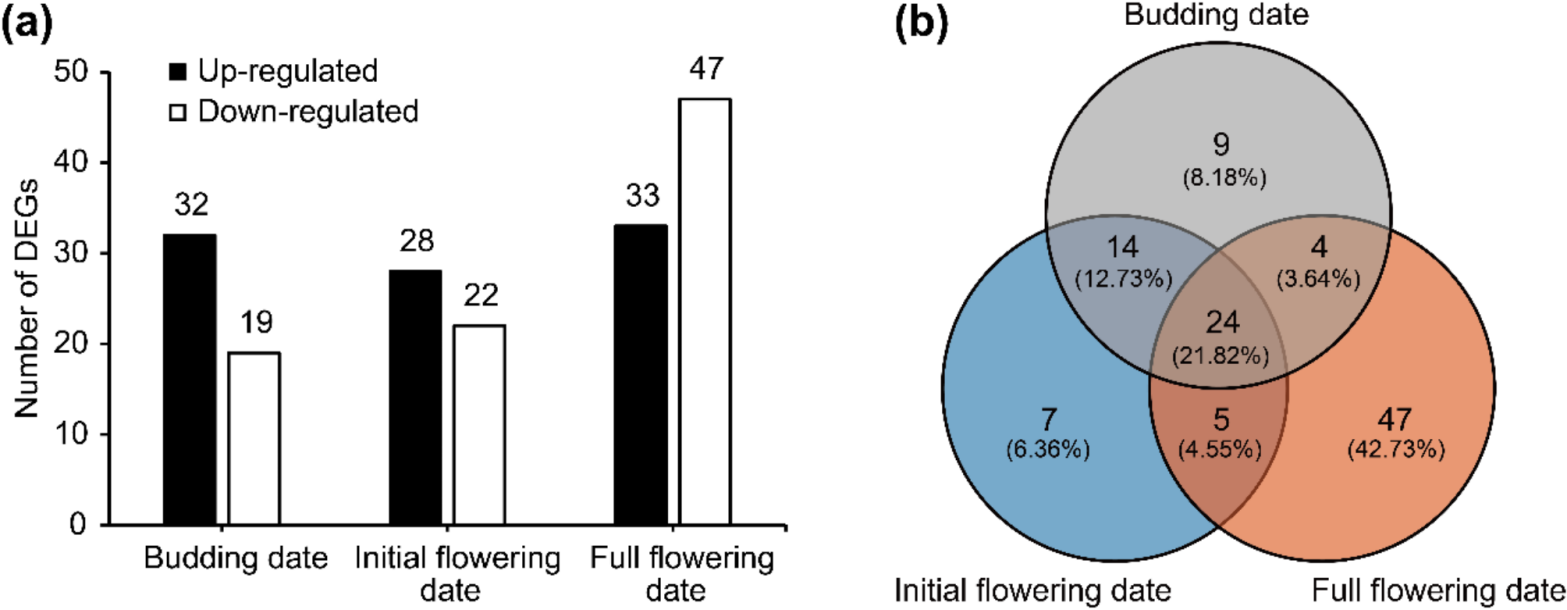
Differential gene expression analysis. (**a**) Up and down regulated DEGs in racemes of both parents at different stages within two QTL clusters; (**b**) Venn diagram of union DEGs in racemes of both parents within two QTL clusters

Within *QTL-cluster1* and *QTL-cluster2*, there were 51, 40 and 80 DEGs associated with BD, IFD and FFD respectively (Fig. 3a). And a total of 110 different DEGs (including 24 common DEGs) were found (Fig. 3b).

**Fig. 4:**
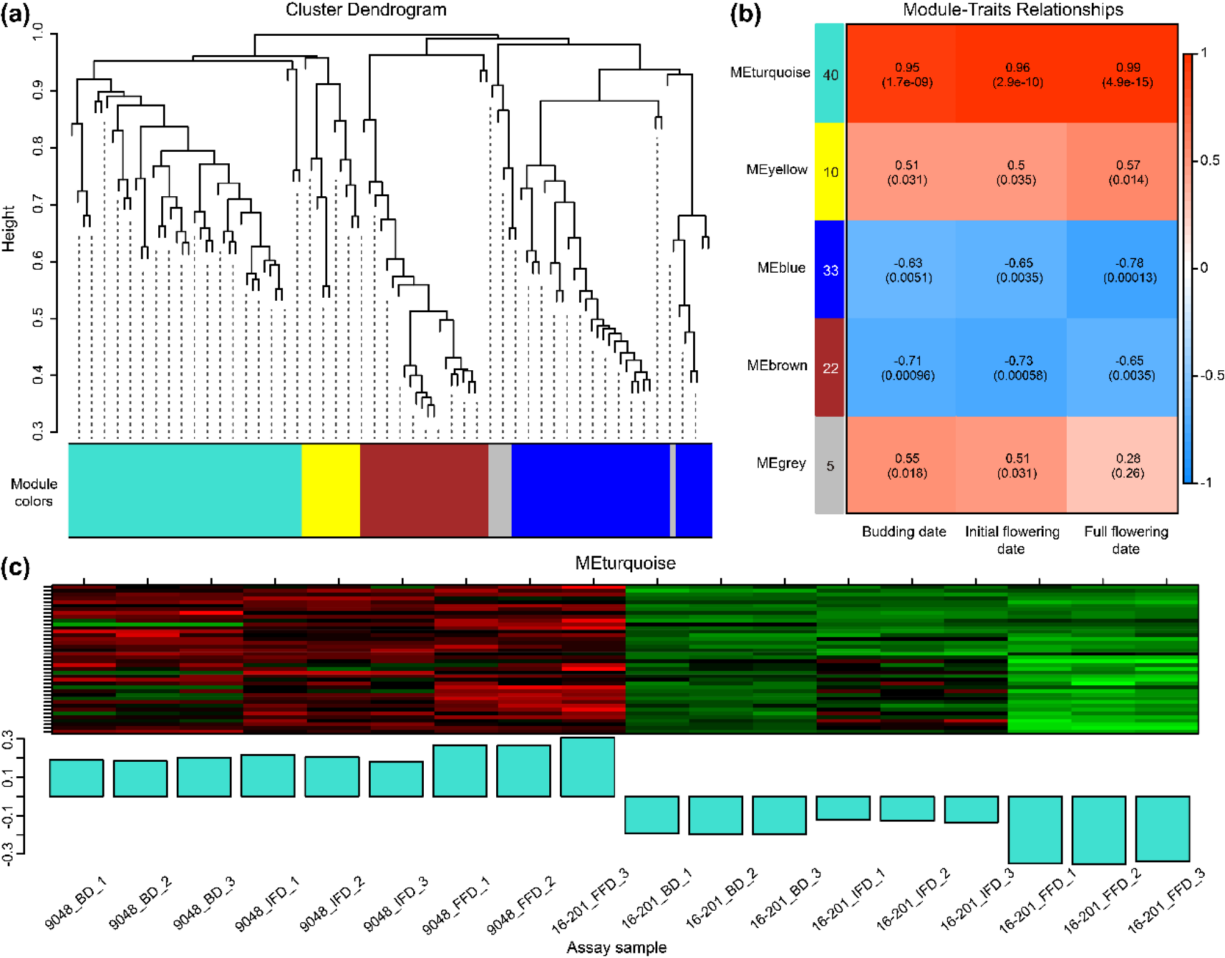
WGCNA of the DEGs expression matrix. (**a**) Gene-based co-expression network analysis dendrogram; (**b**) The correlation heat map between five modules and three stages. Each row represents a module labeled with the same color as in (**a**), the number in each cell represents the correlation coefficient and shows with color, the p-value of the corresponding module-trait is exhibited in parentheses; (**c**) The expression pattern diagram of DEGs in MEturquoise module. The upper part shows the clustering heatmap of DEGs, with high expression in red and low expression in green, and the lower part shows the expression patterns of DEGs in different assay samples

WGCNA on the above 110 DEGs produced five distinct gene co-expression modules (Fig. 4a). Each module was consisted of five to 40 DEGs (Fig. 4b). Among them, the MEturquoise module was significantly correlated with all sampling stages (i.e., BD, IFD and FFD) with correlation coefficients more than 0.95 (*p* < 0.01) (Fig. 4b), which implied the existence of genes controlling castor flowering in this module. Furthermore, 40 DEGs in the MEturquoise module were generally high-expressed in 9048, but low-expressed in 16–201 (Fig. 4c), suggesting that the target genes negatively regulated castor flowering.

### Candidate gene prediction and their relative expression analysis

Combining the annotation information and the available literature descriptions, four candidate genes were screened from 40 DEGs in the MEturquoise module (Fig. S4, Supplementary Table S6). Among them, two genes (viz., *LOC8261128* and *LOC8278994*) in *QTL-cluster1* were annotated as sister chromatid cohesion 1 protein 3 (*RcSYN3*) and zinc finger protein WIP2 (*RcNTT*) respectively, which were predicted to regulate the meiotic progression of male and female gametophytes [49–51] and affect the expression of other flowering genesis factors [52, 53] respectively; the other two genes (viz., *LOC8281165* and *LOC8259049*) in *QTL-cluster2* were annotated as guanine nucleotide-binding protein subunit gamma 3 (*RcGG3*) and auxin-responsive protein SAUR76 (*RcSAUR76*) respectively, which were predicted to control growth stages [54, 55] and mediate plant growth [56] respectively.

**Fig. 5:**
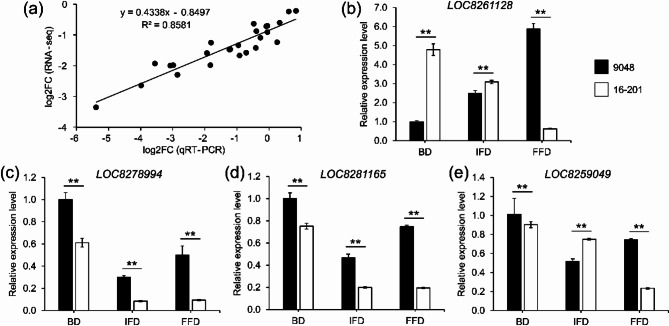
Differential expression of candidate genes controlling growth stages in 9048 and 16–201. (**a**) Correlation between qRT-PCR and RNA-seq data; **(b-e**) Relative expression levels of 4 predicted candidate genes. One-way ANOVA was performed, and ** indicates significance level at 0.01

A total of 11 DEGs (including the above candidate genes and randomly selected DEGs) were selected to conduct qRT-PCR for verifying the availability of the transcriptome data. The qRT-PCR results and RNA-seq results of these 11 DEGs displayed a linear regression trend [i.e., log2FC (qRT-PCR) − 0.8497 = log2FC (RNA-seq), R^2^ = 0.8581], proving that the transcriptome data were reliable (Fig. 5a). The four candidate genes, *LOC8261128*, *LOC8278994*, *LOC8281165* and *LOC8259049*, were significantly differentially expressed (*p* < 0.01) between parents based on qRT-PCR (Fig. 5b-e) and generally high-expressed in 9048 (especially at FFD). The expression differences at BD, IFD and FFD ranged from 1.24 to 9.41, 1.64–5.31, 1.33–3.84 and 1.12–3.19 times respectively.

## Discussion

### Genetic structure of growth stages in castor

Understanding the genetic basis of growth stages in castor will be beneficial for improving yields, reducing costs and breeding varieties adapted to various growing environments and farming systems. In this study, one to four QTLs underlying each trait were detected in three linkage groups (Fig. 2; Table 2, Fig. S3). The PVE of most QTLs was less than 5%, a few QTLs between 5% and 10%, and only two QTLs (i.e., *FqED6.3* and *BqPSMD6.1*) reached 25.46% and 10.82% respectively. Obviously, ED, PSBD, PSFD, PSMD and PBSMD were quantitative traits jointly controlled by major genes and polygene, consistent to the phenotypic genetic analysis results (Fig. 1; Tables 1 and 3). In the future, QTL analysis should be carried out in multi-populations and/or multi-environments using high-density genetic map for further validation of the above identified QTLs and detecting more novel QTLs; after all, QTL mapping was only performed in two segregating populations for one year in this study, and partial results were obtained with the aim of providing a reference for further research and breeding. Here, two advantages arose from the mapping results which may be applied in breeding, one is the molecular marker assisted selection of the two main effect QTLs (*FqED6.3* and *BqPSMD6.1*) to grasp the principal contradiction among numerous genes, another is the genetically related selection using the linkage between the QTLs conferring the same trait and different traits to realize early growth, rapid development and early maturing. As for the epistatic effect, it was the important genetic component of ED, PSBD, PSFD, PSMD and PBSMD in castor, there is not yet a clear way to use it in breeding now (Table 4), mainly because of its complex interaction pattern and the serious interference from environmental factors, which makes it difficult to grasp precisely in breeding [57].

### QTL clusters underlying growth stages in castor

In most cases, the detected QTLs cannot be simultaneously identified in multiple environments [42, 58], which raised concerns about the feasibility of studying them in depth. QTL cluster is favored for genetic manipulation, it contains target genes (at least indicates that the allelic QTLs are reliable), because it consists of the allelic QTLs conferring multiple traits [42] or one trait in multiple environments [58] or the multiple traits in multiple environments [42]. Fortunately, two QTL clusters (i.e., *QTL-cluster1* and *QTL-cluster2*) were found in this study (Fig. 2). *QTL-cluster1*, located in the marker interval RCM1769-RCM1838 (10,477.5 kb), contained most of the detected QTLs, including 2 main-effect QTLs, in populations F_2_ and BC_1_ (Fig. 2; Table 3). It is worth focusing on this region to carry out in-depth and detailed gene mining.

In order to reach certain goals such as flower synchronization and simultaneous harvesting, it is sometimes not essential to significantly change FD and MD in local cultivars. Hence, the genes with minor effects are equally desirable targets for fine-tuning FD and MD in castor [34]. *QTL-cluster2*, located in the marker interval RCM950-RCM917 (936.8 kb) (Fig. 2; Table 3), was consisted of six allelic QTLs underlying PSBD, PSFD, PSMD and PBSMD with each of contribution rate ranged from 3.25 to 7.71% (Table 2), which functioned as either minor effect QTLs to modify the major gene or sub-major effect QTLs to participate in the forming of growth stages.

### Strategy for mining candidate genes

With the decrease of sequencing cost, integrating QTL mapping with other omics analysis (especially transcriptome analysis) has been widely used in candidate gene mining. WGCNA is one of the most popular methods to mine hub factors, which analyzes gene expression patterns with transcriptomic data to construct core gene networks [59]. In this study, four candidate genes were screened out from two QTL clusters with this strategy, combined with annotation information and available literature descriptions. In the same way, the candidate genes regulating root development and fiber development were mined from the corresponding QTL clusters in rapeseed [60, 61] and cotton [62] respectively, which proved the effectiveness of this strategy.

### Candidate gene annotation

In this study, the four candidate genes, viz., *LOC8261128*, *LOC8278994*, *LOC8281165* and *LOC8259049*, were annotated as *RcSYN3*, *RcNTT*, *RcGG3* and *RcSAUR76* respectively (Supplementary Table S6). *AtSYN3* is an essential gene concerning the development of male and female gametophytes in *Arabidopsis*, if knocked out, the meiotic progression will be repressed and the flowering will be delayed in turn [49–51]. It is inferred that *LOC8261128* (*RcSYN3*) delays the flowering of castor plant in the same way. *NO TRANSMITTING TRACT* (*AtNTT*) represses the expression of *FRUITFULL* (*FUL*) [52], which cooperates with *APETALA1* (*AP1*) and *CAULIFLOWER* (*CAL*) to induce *Arabidopsis* flowering [53]. Likewise, *LOC8278994* (*RcNTT*) is inferred to delay FD of castor plant by repressing the expression of positive flowering regulators. The overexpression of *Arabidopsis Guanine nucleotide-binding protein subunit gamma 3* (*AtGG3*) will shorten the vegetative growth and reproductive growth periods [54, 55], which implies that *LOC8281165* (*RcGG3*) functions in controlling the growth stages in castor. In *Arabidopsis*, *AtSAUR76* controls leaf and root development and regulates plant growth by mediating cell number [56]. *LOC8259049* (*RcSAUR76*) is annotated as auxin-responsive protein SAUR76, homologous to *AtSAUR76*, and may function to regulate castor plant growth. Anyway, the significant differential expression between parents remains the important basis for the identification of candidate genes (Fig. 5b-e).

## Conclusion

Dozens of QTLs and four candidate genes (i.e., *LOC8261128*, *LOC8278994*, *LOC8281165* and *LOC8259049*) conferring castor ED, PSBD, PSFD, PSMD and PBSMD were identified in populations F_2_ and BC_1_. However, the real function of these candidate genes has not been determined. Therefore, in the future, it is necessary to analyze whether these four genes affect castor FD through molecular biology technologies (such as gene overexpression and RNA interference), and explore their exact molecular mechanisms. It is conducive to the breeding of superior castor varieties with high yield and early maturity, and introduces them to high latitude regions.

## Electronic supplementary material

Below is the link to the electronic supplementary material.

Supplementary Material 1

Supplementary Material 2

Supplementary Material 3

Supplementary Material 4

Supplementary Material 5

Supplementary Material 6

Supplementary Material 7

Supplementary Material 8

Supplementary Material 9

Supplementary Material 10

## Data Availability

The data that support the findings of this study are available from the corresponding author Xuegui Yin on reasonable request. Transcriptome sequencing data have been deposited in the Sequence Read Archive (SRA) under accession number PRJNA1105697.

## Abbreviations

QTL: Quantitative trait locus
CIM: Composite interval mapping
ICIM: Inclusive composite interval mapping
PVE: Phenotypic variation explained
PCR: Polymerase chain reaction
qRT-PCR: Quantitative real-time polymerase chain reaction
ORFs: Open reading frames
ED: Emergence date
PSBD: Budding date of primary spike
PSFD: Flowering date of primary spike
PSMD: Maturation date of primary spike
PBSMD: Maturation date of primary branch spike
DAS: Days after sowing
BD: Budding date
IFD: Initial flowering date
FFD: Full flowering date
LG: Linkage group
Add.: Additive effect
Dom.: Dominance effect
Pos.: Position
CI: Confidence interval
MI: Marker interval
