# Supplementary material for: Integrating QTL mapping with transcriptome analysis mined candidate genes of growth stages in castor (Ricinus communis L.)
Source: BMC Genomics. 2025 Feb 22;26:178. doi: 10.1186/s12864-025-11348-9 (PMC11846381; doi:10.1186/s12864-025-11348-9)
Supplement: Supplementary file 1 — Supplementary Material 1 [file 12864_2025_11348_MOESM1_ESM.docx]

**Fig. S1** Number of stem nodes in both parents. Each solid arrow and dashed arrow indicate a node in primary stem and primary branch stem respectively; Bars for 10 cm
