# Supplementary material for: Integrating QTL mapping with transcriptome analysis mined candidate genes of growth stages in castor (Ricinus communis L.)
Source: BMC Genomics. 2025 Feb 22;26:178. doi: 10.1186/s12864-025-11348-9 (PMC11846381; doi:10.1186/s12864-025-11348-9)
Supplement: Supplementary file 2 — Supplementary Material 2 [file 12864_2025_11348_MOESM2_ESM.docx]

**Fig.S2** Distribution of 566 pairs of SSR primers on the castor genome ASM1957865v1.
