# Supplementary material for: Integrating QTL mapping with transcriptome analysis mined candidate genes of growth stages in castor (Ricinus communis L.)
Source: BMC Genomics. 2025 Feb 22;26:178. doi: 10.1186/s12864-025-11348-9 (PMC11846381; doi:10.1186/s12864-025-11348-9)
Supplement: Supplementary file 3 — Supplementary Material 3 [file 12864_2025_11348_MOESM3_ESM.docx]

**Supplementary Table S1** Primer sequences of genes

| **Gene** | **Sequence (5′-3′)** | **Product size (bp)** |
| --- | --- | --- |
| *GAPDH* | F: CTCCCATGTTTGTTGTTGGTGTCA | 217 |
|  | R: CTTCCACCTCTCCAGTCCTTCATT |  |
| *LOC8261128* | F: GAATGCAGGAATCAGGCCAAC | 83 |
|  | R: TAGGAATGGTATGCGGGGAAG |  |
| *LOC8281165* | F: CAAGCGCAGAGAAATGGCTA | 144 |
|  | R: GGATCTGAATTTGCCACCACA |  |
| *LOC8278994* | F: TCATGTCCTGTTTGCCTCAAGA | 127 |
|  | R: TTAGCATTGCTGTTGGTTGGG |  |
| *LOC8259049* | F: GCCAAAGGAGGCAAGCTAAC | 122 |
|  | R: GAGGTGGAGGAGTCATCAGC |  |
| *LOC8258322* | F: AGGCCAGGACACCTAGAAATG | 96 |
|  | R: CAGGCACAAGTGGAATAGGGT |  |
| *LOC8260886* | F: CGTGCCCAAGGTGTGATTC | 146 |
|  | R: GGTTCCACCGCCAATAGGTA |  |
| *LOC8264789* | F: CCGGGACCAGAGCTAAAGAC | 107 |
|  | R: CACATTCCCTGTTGGAGCCT |  |
| *LOC8265222* | F: AACGCACAATGCCTTAGCAAT | 145 |
|  | R: CCCTGGCTTCTCTGTCAACT |  |
| *LOC8266790* | F: ACCACAGGCGACATAAGGTT | 136 |
|  | R: CAGCTCCTCTTTGCTTCGTC |  |
| *LOC8266867* | F: AGGGTTGAGCATGGAAGAACT | 135 |
|  | R: TCCATCAATTCAGCTCCCTTTCT |  |
| *LOC8269211* | F: GCGACGCTGAAGTTTGCTTA | 137 |
|  | R: TCTGGTACGCAGGTCAGTTG |  |
| *LOC8277502* | F: TCAGCTAGGCCACAAGAACAA | 101 |
|  | R: TGTCTTGGCTGTGTTAGACTGT |  |
| *LOC8285795* | F: CTCCTTGGACTGACGGAACC | 87 |
|  | R: GGTGAAATTCAGCTCCGCAAG |  |
