# Supplementary material for: Integrating QTL mapping with transcriptome analysis mined candidate genes of growth stages in castor (Ricinus communis L.)
Source: BMC Genomics. 2025 Feb 22;26:178. doi: 10.1186/s12864-025-11348-9 (PMC11846381; doi:10.1186/s12864-025-11348-9)
Supplement: Supplementary file 4 — Supplementary Material 4 [file 12864_2025_11348_MOESM4_ESM.docx]

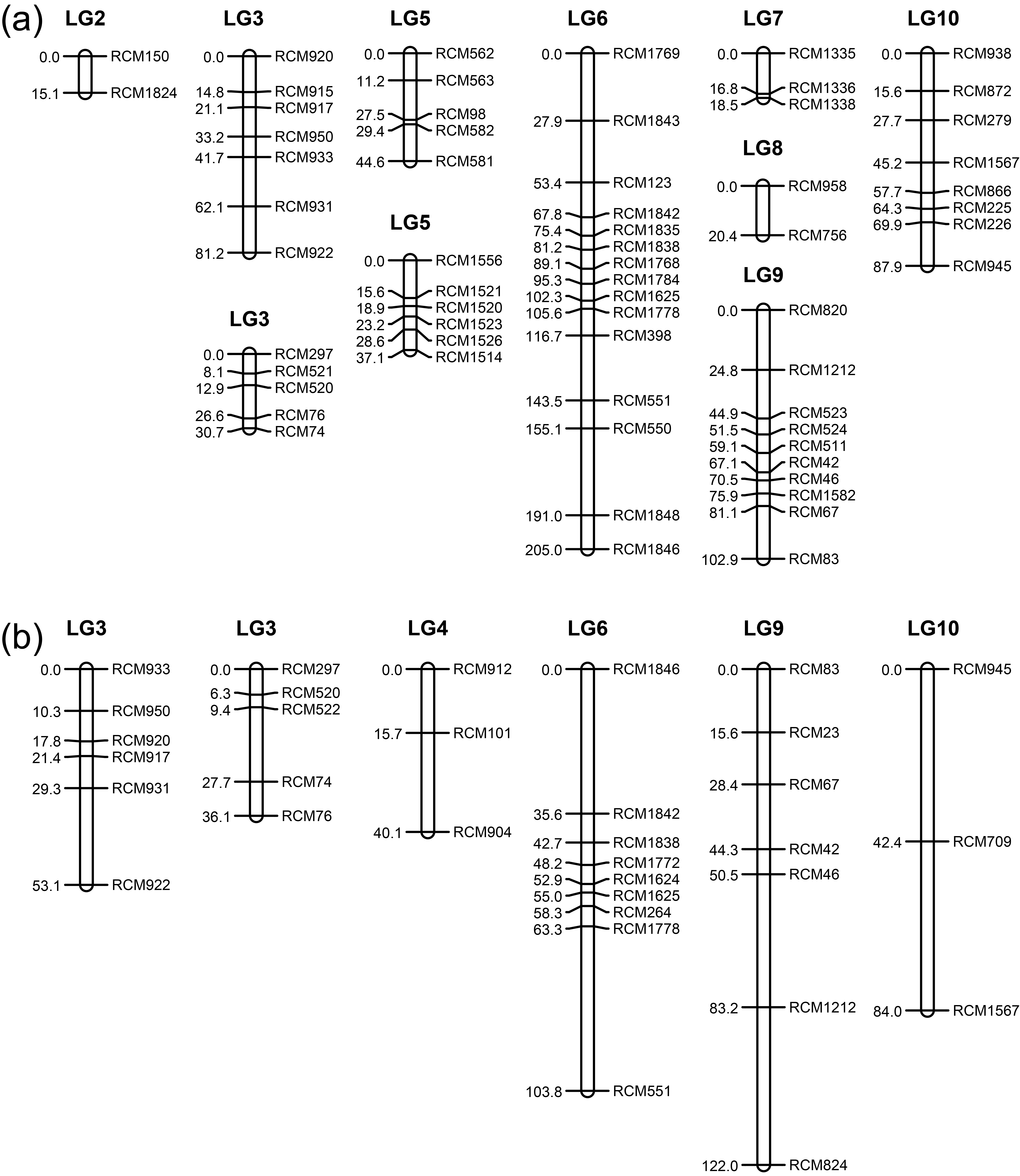


**Fig. S3** The genetic linkage map in castor plants. (**a**) and (**b**) were the genetic maps constructed in populations F_2_ and BC_1_ respectively
