# Supplementary material for: Integrating QTL mapping with transcriptome analysis mined candidate genes of growth stages in castor (Ricinus communis L.)
Source: BMC Genomics. 2025 Feb 22;26:178. doi: 10.1186/s12864-025-11348-9 (PMC11846381; doi:10.1186/s12864-025-11348-9)
Supplement: Supplementary file 5 — Supplementary Material 5 [file 12864_2025_11348_MOESM5_ESM.docx]

**Supplementary Table S2** Identification of epistasis QTL conferring growth stages in F_2_ population

| **Trait** | **QTL_1** | **LG**  **_1** | **Pos.**  **_1** | **MI_1** | **QTL_2** | **LG**  **_2** | **Pos.**  **_2** | **MI_2** | **LOD** | **PVE**  **(%)** | **Add.**  **_1** | **Add.**  **_2** | **Dom.**  **_1** | **Dom.**  **_2** | **AA** | **AD** | **DA** | **DD** |
| --- | --- | --- | --- | --- | --- | --- | --- | --- | --- | --- | --- | --- | --- | --- | --- | --- | --- | --- |
| ED | ***FqED6.1*** | 6 | 15 | RCM1769-RCM1843 | ***FqED6.2*** | 6 | 175 | RCM550-RCM1848 | 61.92 | 6.98 | 0.79 | -1.82 | -1.73 | -1.83 | -0.63 | -0.68 | 1.72 | 3.59 |
|  | *FqED9.1* | 9 | 20 | RCM820-RCM1212 | *FqED9.2* | 9 | 30 | RCM1212-RCM523 | 28.08 | 6.66 | 0.86 | -0.82 | -0.77 | 0.25 | -1.73 | -0.94 | -0.07 | -1.22 |
|  | ***FqED6.1*** | 6 | 15 | RCM1769-RCM1843 | *FqED3.1* | 3 | 50 | RCM933-RCM931 | 13.63 | 6.13 | -0.50 | 0.58 | 0.41 | 0.44 | -0.53 | 1.54 | -1.46 | -1.36 |
|  | ***FqED6.1*** | 6 | 10 | RCM1769-RCM1843 | *FqED9.4* | 9 | 90 | RCM67-RCM83 | 9.80 | 6.05 | -0.96 | -0.01 | -0.98 | -0.06 | 0.03 | 2.04 | 0.07 | -0.02 |
|  | *FqED9.4* | 9 | 100 | RCM67-RCM83 | *FqED5.2* | 5 | 40 | RCM582-RCM581 | 9.55 | 2.11 | 0.08 | -0.18 | 2.25 | 0.27 | 0.00 | -0.11 | -2.12 | -2.42 |
|  | *FqED3.1* | 3 | 45 | RCM933-RCM931 | *FqED3.1* | 3 | 50 | RCM933-RCM931 | 9.34 | 3.26 | -0.37 | 0.55 | 2.75 | 0.23 | -0.26 | -0.52 | 1.06 | -3.09 |
|  | *FqED5.2* | 5 | 40 | RCM582-RCM581 | *FqED3.2* | 3 | 75 | RCM931-RCM922 | 9.21 | 2.35 | -0.87 | 0.85 | -0.16 | -0.63 | -0.74 | 0.79 | -1.20 | 0.19 |
|  | ***FqED6.1*** | 6 | 10 | RCM1769-RCM1843 | *FqED5.2* | 5 | 35 | RCM582-RCM581 | 8.90 | 3.07 | 0.80 | -1.69 | -1.62 | -0.96 | -0.60 | -1.15 | 1.75 | 1.10 |
|  | *FqED10.2* | 10 | 20 | RCM872-RCM279 | *FqED10.3* | 10 | 30 | RCM279-RCM1567 | 7.39 | 1.51 | -0.13 | 0.11 | -2.38 | -2.37 | -2.12 | 0.28 | 0.05 | 2.63 |
|  | *FqED5.2* | 5 | 30 | RCM582-RCM581 | *FqED5.2* | 5 | 40 | RCM582-RCM581 | 7.08 | 2.16 | -0.50 | 0.34 | 2.40 | -0.25 | -0.32 | 0.46 | -2.17 | -2.29 |
|  | *FqED7.1* | 7 | 10 | RCM1335-RCM1336 | *FqED10.4* | 10 | 55 | RCM1567-RCM866 | 6.85 | 1.22 | -0.42 | 0.18 | -0.07 | 0.05 | -0.23 | 1.13 | -0.22 | -0.15 |
|  | *FqED10.2* | 10 | 20 | RCM872-RCM279 | *FqED3.3* | 3 | 25 | RCM520-RCM76 | 6.79 | 1.90 | 0.87 | 0.99 | -0.97 | -0.95 | 1.07 | -0.86 | -1.03 | 0.94 |
|  | *FqED9.5* | 9 | 30 | RCM1212-RCM523 | *FqED10.1* | 10 | 10 | RCM938-RCM872 | 6.56 | 2.43 | 1.38 | 0.76 | -1.33 | -1.02 | 1.13 | -1.63 | -0.85 | 1.09 |
|  | *FqED5.2* | 5 | 40 | RCM582-RCM581 | *FqED3.4* | 3 | 10 | RCM521-RCM520 | 6.54 | 1.32 | -0.59 | 0.43 | -0.06 | -0.31 | -0.57 | 0.66 | -0.52 | 0.19 |
|  | ***FqED6.1*** | 6 | 10 | RCM1769-RCM1843 | *FqED10.2* | 10 | 20 | RCM872-RCM279 | 6.48 | 3.06 | 0.51 | 0.32 | -0.66 | -0.05 | 1.00 | -1.26 | -0.27 | -0.12 |
|  | *FqED3.3* | 3 | 15 | RCM520-RCM76 | *FqED3.3* | 3 | 20 | RCM520-RCM76 | 6.43 | 2.43 | 0.41 | -0.39 | -0.61 | 2.62 | -0.19 | 1.17 | 0.45 | -2.27 |
|  | *FqED8.1* | 8 | 0 | RCM958-RCM756 | *FqED8.1* | 8 | 15 | RCM958-RCM756 | 6.22 | 1.29 | 1.25 | -1.42 | -0.90 | -1.27 | -0.94 | -1.05 | 1.20 | 1.30 |
|  | *FqED10.2* | 10 | 25 | RCM872-RCM279 | *FqED5.2* | 5 | 40 | RCM582-RCM581 | 6.06 | 2.15 | 1.13 | -1.25 | -1.33 | -1.11 | -0.90 | -1.22 | 1.09 | 1.40 |
|  | *FqED3.5* | 3 | 0 | RCM297-RCM521 | *FqED3.2* | 3 | 70 | RCM931-RCM922 | 6.05 | 2.28 | 0.58 | 0.44 | -0.91 | -1.26 | 0.96 | -0.56 | -0.42 | 1.12 |
|  | *FqED3.5* | 6 | 15 | RCM1769-RCM1843 | *FqED3.3* | 3 | 20 | RCM520-RCM76 | 6.01 | 2.43 | 0.62 | 1.39 | -0.98 | -1.06 | 0.63 | -0.62 | -1.75 | 0.86 |
|  | *FqED5.2* | 5 | 35 | RCM582-RCM581 | *FqED8.1* | 8 | 5 | RCM958-RCM756 | 5.89 | 1.74 | -0.85 | 0.43 | -0.88 | -1.04 | -0.85 | 0.65 | -0.49 | 1.37 |
|  | *FqED7.1* | 7 | 0 | RCM1335-RCM1336 | *FqED9.3* | 9 | 45 | RCM523-RCM524 | 5.44 | 0.71 | -0.41 | -0.62 | -0.60 | -0.68 | 1.09 | 0.58 | 0.54 | 0.55 |
|  | *FqED5.1* | 5 | 10 | RCM1556-RCM1521 | *FqED5.1* | 5 | 15 | RCM1556-RCM1521 | 5.37 | 1.54 | 0.17 | -0.14 | 0.00 | 2.32 | 0.08 | 1.93 | -0.24 | -2.31 |
|  | ***FqED6.1*** | 6 | 10 | RCM1769-RCM1843 | *FqED8.1* | 8 | 10 | RCM958-RCM756 | 5.15 | 2.93 | -0.49 | -0.46 | -0.32 | 0.66 | 0.37 | 1.31 | 0.54 | -0.72 |
|  | *FqED10.5* | 10 | 75 | RCM226-RCM945 | *FqED3.2* | 3 | 75 | RCM931-RCM922 | 5.15 | 1.79 | 1.20 | 0.97 | -1.08 | -1.33 | 1.04 | -1.38 | -1.14 | 1.07 |
| PSBD | ***FqPSBD6.1*** | 6 | 15 | RCM1769-RCM1843 | *FqPSBD2.1* | 2 | 15 | RCM150-RCM1824 | 5.54 | 8.88 | 0.05 | -2.50 | 0.60 | -1.89 | 2.55 | -7.02 | 3.96 | 4.83 |
| PSFD | *FqPSFD6.4* | 6 | 175 | RCM550-RCM1848 | *FqPSFD6.4* | 6 | 190 | RCM550-RCM1848 | 6.71 | 12.40 | 4.72 | -5.14 | -12.45 | -5.42 | 3.14 | -13.69 | 4.29 | 18.14 |
|  | ***FqPSFD6.2*** | 6 | 15 | RCM1769-RCM1843 | *FqPSFD2.1* | 2 | 10 | RCM150-RCM1824 | 6.53 | 10.25 | 2.91 | -4.26 | -2.85 | -4.88 | 3.84 | -11.13 | 7.78 | 10.77 |
|  | ***FqPSFD6.2*** | 6 | 15 | RCM1769-RCM1843 | *FqPSFD10.1* | 10 | 35 | RCM279-RCM1567 | 5.38 | 12.24 | -4.27 | 3.10 | -2.47 | -7.81 | 3.80 | -1.25 | -9.55 | 15.38 |
| PSMD | *FqPSMD6.1* | 6 | 15 | RCM1769-RCM1843 | *FqPSMD6.2* | 6 | 60 | RCM123-RCM1842 | 65.18 | 11.39 | -2.69 | -2.70 | 4.18 | 5.33 | 2.70 | 4.24 | 9.55 | -12.21 |
|  | *FqPSMD6.1* | 6 | 10 | RCM1769-RCM1843 | *FqPSMD9.1* | 9 | 15 | RCM820-RCM1212 | 42.17 | 11.21 | -2.06 | -3.65 | -8.75 | -8.75 | -3.05 | 2.06 | 3.64 | 15.45 |
|  | *FqPSMD7.1* | 7 | 5 | RCM1335-RCM1336 | *FqPSMD6.1* | 6 | 10 | RCM1769-RCM1843 | 29.48 | 10.75 | 0.68 | -5.19 | 3.67 | -5.20 | -0.90 | -0.37 | 7.47 | -3.92 |
|  | *FqPSMD6.1* | 6 | 10 | RCM1769-RCM1843 | *FqPSMD8.1* | 8 | 10 | RCM958-RCM756 | 18.17 | 10.23 | 2.32 | -3.07 | 0.72 | 2.39 | -2.32 | -7.57 | 6.56 | -6.12 |
| PBSMD | *FqPBSMD6.4* | 6 | 20 | RCM1769-RCM1843 | *FqPBSMD6.5* | 6 | 170 | RCM550-RCM1848 | 27.15 | 18.74 | -5.14 | -0.55 | -5.36 | -0.38 | 0.04 | 10.29 | 0.33 | 10.67 |

LG, Pos., Add., Dom., CI and MI are abbreviations for linkage group, position, additive effect, dominance effect, confidence interval and marker interval respectively

“-” indicates a missing value; the underlined loci have both epistatic and single-locus effects

The trait description is the same as in Table 1
