# Supplementary material for: Integrating QTL mapping with transcriptome analysis mined candidate genes of growth stages in castor (Ricinus communis L.)
Source: BMC Genomics. 2025 Feb 22;26:178. doi: 10.1186/s12864-025-11348-9 (PMC11846381; doi:10.1186/s12864-025-11348-9)
Supplement: Supplementary file 6 — Supplementary Material 6 [file 12864_2025_11348_MOESM6_ESM.docx]

**Supplementary Table S3** Identification of epistasis QTL conferring growth stages in BC_1_ population

| **Trait** | **QTL_1** | **LG_1** | **Pos._1** | **MI_1** | **QTL_2** | **LG_2** | **Pos._2** | **MI_2** | **LOD** | **PVE**  **(%)** | **Add._1** | **Add._2** | **AA** |
| --- | --- | --- | --- | --- | --- | --- | --- | --- | --- | --- | --- | --- | --- |
| ED | *BqED9.1* | 9 | 70 | RCM46-RCM1212 | *BqED9.2* | 9 | 90 | RCM1212-RCM824 | 14.88 | 4.02 | -1.43 | -4.59 | -5.93 |
|  | *BqED10.1* | 10 | 40 | RCM945-RCM709 | *BqED10.2* | 10 | 50 | RCM709-RCM1567 | 13.74 | 3.44 | -4.43 | 0.26 | -4.18 |
| PSMD | *BqPSMD9.1* | 9 | 70 | RCM46-RCM1212 | *BqPSMD9.2* | 9 | 90 | RCM1212-RCM824 | 21.62 | 3.50 | -9.17 | -9.69 | -19.21 |
|  | *BqPSMD10.1* | 10 | 65 | RCM709-RCM1567 | *BqPSMD10.1* | 10 | 70 | RCM709-RCM1567 | 21.50 | 3.54 | -9.96 | 0.00 | -9.04 |
|  | ***BqPSMD6.1*** | 6 | 85 | RCM1778-RCM551 | ***BqPSMD6.1*** | 6 | 90 | RCM1778-RCM551 | 19.50 | 3.50 | -9.50 | 0.69 | -8.74 |
|  | ***BqPSMD3.1*** | 3 | 45 | RCM931-RCM922 | *BqPSMD10.2* | 10 | 15 | RCM945-RCM709 | 6.80 | 3.32 | 0.08 | -9.10 | -7.99 |

LG, Pos., Add., CI and MI are abbreviations for linkage group, position, additive effect, confidence interval and marker interval respectively

“-” indicates a missing value; the underlined loci have both epistatic and single-locus effects

The trait description is the same as in Table 1
