# Supplementary material for: Integrating QTL mapping with transcriptome analysis mined candidate genes of growth stages in castor (Ricinus communis L.)
Source: BMC Genomics. 2025 Feb 22;26:178. doi: 10.1186/s12864-025-11348-9 (PMC11846381; doi:10.1186/s12864-025-11348-9)
Supplement: Supplementary file 7 — Supplementary Material 7 [file 12864_2025_11348_MOESM7_ESM.docx]

**Supplementary Table S4** Transcriptome sequencing outputs and statistics

| **Assay sample** | **Raw reads** | **Clean reads** | **Clean base (G)** | **Error rate (%)** | **Q20 (%)** | **Q30 (%)** | **GC content (%)** |
| --- | --- | --- | --- | --- | --- | --- | --- |
| 9048_BD_1 | 52,364,710 | 49,708,540 | 7.46 | 0.01 | 98.88 | 96.63 | 43.31 |
| 9048_BD_2 | 73,172,014 | 69,899,930 | 10.48 | 0.01 | 98.89 | 96.65 | 43.34 |
| 9048_BD_3 | 65,379,884 | 62,532,064 | 9.38 | 0.01 | 98.83 | 96.47 | 43.38 |
| 9048_IFD_1 | 69,147,490 | 66,379,650 | 9.96 | 0.01 | 98.9 | 96.67 | 43.46 |
| 9048_IFD_2 | 50,450,394 | 48,915,670 | 7.34 | 0.01 | 98.84 | 96.49 | 43.55 |
| 9048_IFD_3 | 59,174,826 | 56,745,646 | 8.51 | 0.01 | 98.85 | 96.54 | 43.53 |
| 9048_FFD_1 | 64,936,890 | 61,681,644 | 9.25 | 0.01 | 98.73 | 96.24 | 43.75 |
| 9048_FFD_2 | 51,940,680 | 49,813,362 | 7.47 | 0.01 | 98.92 | 96.74 | 43.45 |
| 9048_FFD_3 | 50,913,874 | 47,924,180 | 7.19 | 0.01 | 98.92 | 96.75 | 43.46 |
| 16-201_BD_1 | 54,425,414 | 51,589,254 | 7.74 | 0.01 | 98.97 | 96.89 | 43.24 |
| 16-201_BD_2 | 49,829,290 | 46,434,230 | 6.97 | 0.01 | 98.51 | 95.55 | 43.29 |
| 16-201_BD_3 | 47,298,350 | 45,297,550 | 6.79 | 0.01 | 98.91 | 96.71 | 43.2 |
| 16-201_IFD_1 | 45,007,024 | 42,770,782 | 6.42 | 0.01 | 98.92 | 96.76 | 43.15 |
| 16-201_IFD_2 | 50,717,686 | 48,390,612 | 7.26 | 0.01 | 98.94 | 96.8 | 43.2 |
| 16-201_IFD_3 | 50,180,810 | 47,616,236 | 7.14 | 0.01 | 98.9 | 96.66 | 43.25 |
| 16-201_FFD_1 | 59,144,682 | 56,626,238 | 8.49 | 0.01 | 98.94 | 96.79 | 43.09 |
| 16-201_FFD_2 | 47,229,758 | 44,987,118 | 6.75 | 0.01 | 98.89 | 96.65 | 43.2 |
| 16-201_FFD_3 | 44,028,306 | 41,724,820 | 6.26 | 0.01 | 98.82 | 96.45 | 43.18 |

BD, IFD and FFD were abbreviations for budding date, initial flowering date and full flowering date respectively
