# Supplementary material for: Integrating QTL mapping with transcriptome analysis mined candidate genes of growth stages in castor (Ricinus communis L.)
Source: BMC Genomics. 2025 Feb 22;26:178. doi: 10.1186/s12864-025-11348-9 (PMC11846381; doi:10.1186/s12864-025-11348-9)
Supplement: Supplementary file 8 — Supplementary Material 8 [file 12864_2025_11348_MOESM8_ESM.docx]

**Supplementary Table S5** Clean reads alignment statistics

| **Assay sample** | **Clean reads** | **Reads mapped** | **Unique mapped** | **Multi mapped** |
| --- | --- | --- | --- | --- |
| 9048_BD_1 | 49,708,540 | 48,551,460 (97.67%) | 47,479,960 (95.52%) | 1,071,500 (2.16%) |
| 9048_BD_2 | 69,899,930 | 68,376,759 (97.82%) | 66,77,7022 (95.53%) | 1,599,737 (2.29%) |
| 9048_BD_3 | 62,532,064 | 61,146,655 (97.78%) | 59,690,328 (95.46%) | 1,456,327 (2.33%) |
| 9048_IFD_1 | 66,379,650 | 64,970,642 (97.88%) | 63,466,765 (95.61%) | 1,503,877 (2.27%) |
| 9048_IFD_2 | 48,915,670 | 47,838,520 (97.80%) | 46,720,178 (95.51%) | 1,118,342 (2.29%) |
| 9048_IFD_3 | 56,745,646 | 55,565,511 (97.92%) | 54,280,051 (95.66%) | 1,285,460 (2.27%) |
| 9048_FFD_1 | 61,681,644 | 59,851,444 (97.03%) | 58,297,490 (94.51%) | 1,553,954 (2.52%) |
| 9048_FFD_2 | 49,813,362 | 48,534,740 (97.43%) | 47,448,157 (95.25%) | 1,086,583 (2.18%) |
| 9048_FFD_3 | 47,924,180 | 46,683,255 (97.41%) | 45,657,451 (95.27%) | 1,025,804 (2.14%) |
| 16-201_BD_1 | 51,589,254 | 50,426,619 (97.75%) | 49,285,595 (95.53%) | 1,141,024 (2.21%) |
| 16-201_BD_2 | 46,434,230 | 45,100,972 (97.13%) | 44,135,016 (95.05%) | 9,659,56 (2.08%) |
| 16-201_BD_3 | 45,297,550 | 44,238,055 (97.66%) | 43,270,927 (95.53%) | 9,671,28 (2.14%) |
| 16-201_IFD_1 | 42,770,782 | 41,786,262 (97.70%) | 40,887,621 (95.60%) | 8,986,41 (2.10%) |
| 16-201_IFD_2 | 48,390,612 | 47,313,226 (97.77%) | 46,299,550 (95.68%) | 1,013,676 (2.09%) |
| 16-201_IFD_3 | 47,616,236 | 46,572,405 (97.81%) | 45,553,280 (95.67%) | 1,019,125 (2.14%) |
| 16-201_FFD_1 | 56,626,238 | 55,373,488 (97.79%) | 54,117,067 (95.57%) | 1,256,421 (2.22%) |
| 16-201_FFD_2 | 44,987,118 | 43,902,138 (97.59%) | 42,926,914 (95.42%) | 9,752,24 (2.17%) |
| 16-201_FFD_3 | 41,724,820 | 40,740,727 (97.64%) | 39,847,762 (95.50%) | 8,929,65 (2.14%) |

BD, IFD and FFD were abbreviations for budding date, initial flowering date and full flowering date respectively
