# Supplementary figures and images for: Integrating QTL mapping with transcriptome analysis mined candidate genes of growth stages in castor (Ricinus communis L.)
Source: BMC Genomics. 2025 Feb 22;26:178. doi: 10.1186/s12864-025-11348-9 (PMC11846381; doi:10.1186/s12864-025-11348-9)

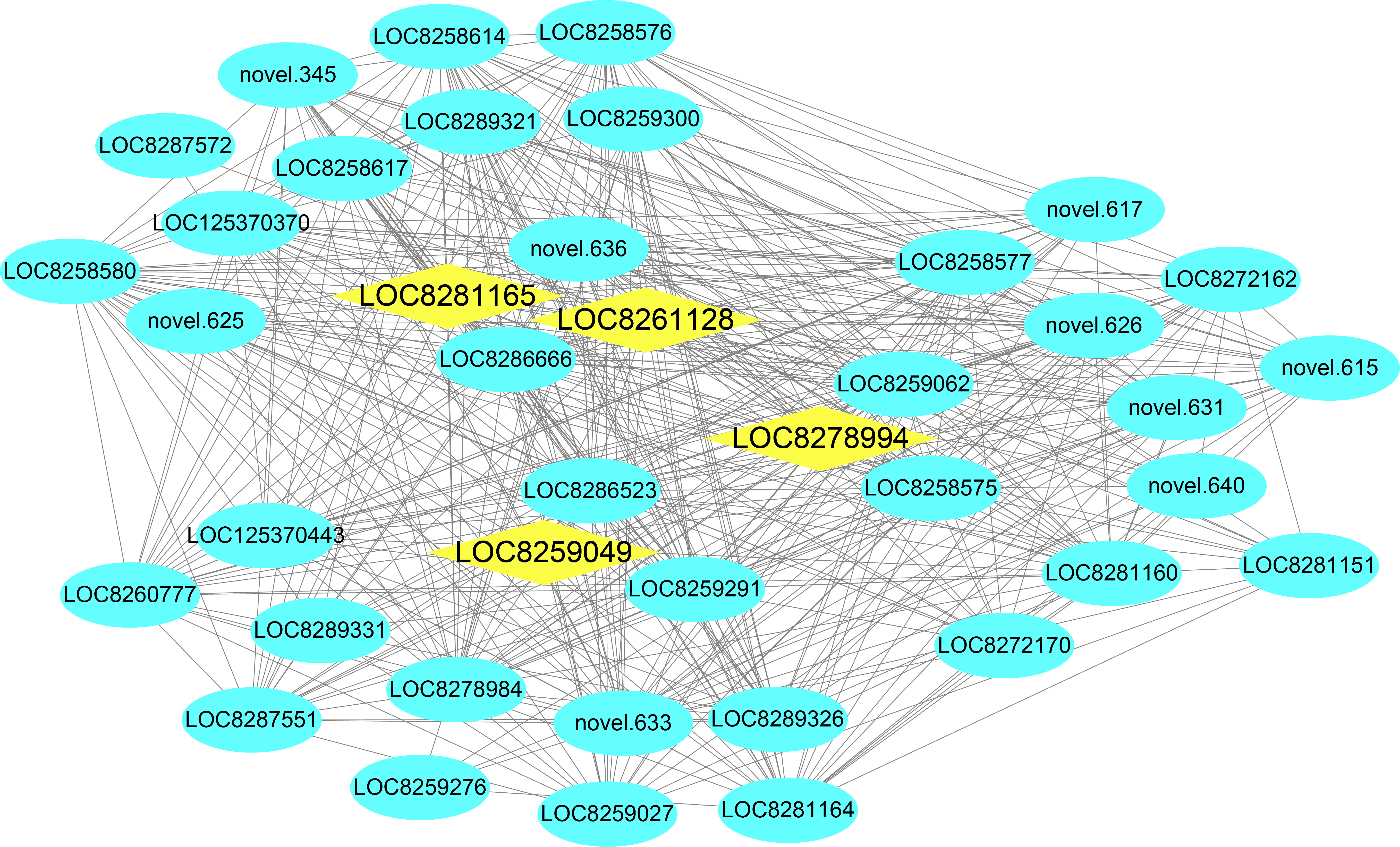


**Fig. S4** Network of DEGs in the MEturquoise module.

Supplement: Supplementary file 9 — Supplementary Material 9 [file 12864_2025_11348_MOESM9_ESM.docx]
