# Supplementary material for: Integrating QTL mapping with transcriptome analysis mined candidate genes of growth stages in castor (Ricinus communis L.)
Source: BMC Genomics. 2025 Feb 22;26:178. doi: 10.1186/s12864-025-11348-9 (PMC11846381; doi:10.1186/s12864-025-11348-9)
Supplement: Supplementary file 10 — Supplementary Material 10 [file 12864_2025_11348_MOESM10_ESM.docx]

**Supplementary Table S6** Gene annotation

| **Gene** | **Chromosome** | **BlastP annotation** | **Swissprot annotation ^a^** |
| --- | --- | --- | --- |
| *LOC8259276* | 6 | GDSL esterase/lipase | GDL1_CARPA |
| *LOC8259291* | 6 | phenylacetaldehyde reductase isoform X1 | PAR1_ROSHC |
| *LOC8259300* | 6 | uncharacterized protein LOC8259300 | -- |
| *LOC8260777* | 6 | malonyl-coenzyme A: anthocyanin 3-O-glucoside-6''-O-malonyltransferase | 3MAT_DAHPI |
| *LOC8261128* | 6 | sister chromatid cohesion 1 protein 3 | SCC13_ARATH |
| *LOC8272162* | 6 | uncharacterized protein LOC8272162 | -- |
| *LOC8272170* | 6 | uncharacterized protein LOC8272170 | -- |
| *LOC8278984* | 6 | putative disease resistance protein RGA4 | RGA1_SOLBU |
| *LOC8278994* | 6 | zinc finger protein WIP2 | ZWIP2_ARATH |
| *LOC8286523* | 6 | ferric reduction oxidase 7, chloroplastic | FRO7_ARATH |
| *LOC8286666* | 6 | O-fucosyltransferase 20 | OFT20_ARATH |
| *LOC8287551* | 6 | uncharacterized protein LOC8287551 | -- |
| *LOC8287572* | 6 | linoleate 13S-lipoxygenase 2-1, chloroplastic | LOX21_SOLTU |
| *LOC8289321* | 6 | AAA-ATPase At4g25835 | AATPE_ARATH |
| *LOC8289326* | 6 | non-specific lipid transfer protein GPI-anchored 14 | LTG14_ARATH |
| *LOC8289331* | 6 | uncharacterized protein LOC8289331 isoform X1 | -- |
| *novel.615* | 6 | uncharacterized protein LOC125371152 | -- |
| *novel.617* | 6 | conserved hypothetical protein | -- |
| *novel.625* | 6 | -- | -- |
| *novel.626* | 6 | uncharacterized protein LOC8277702 isoform X1 | -- |
| *novel.631* | 6 | -- | -- |
| *novel.633* | 6 | conserved hypothetical protein | -- |
| *novel.636* | 6 | -- | -- |
| *novel.640* | 6 | conserved hypothetical protein | -- |
| *LOC125370370* | 6 | conserved hypothetical protein | -- |
| *LOC125370443* | 6 | hypothetical protein RCOM_0968060 | -- |
| *LOC8258575* | 3 | transcription factor WER | MYB2_CROXC |
| *LOC8258576* | 3 | trihelix transcription factor DF1 | TGT2_ARATH |
| *LOC8258577* | 3 | cytochrome P450 72A397 isoform X1 | C7A39_KALSE |
| *LOC8258580* | 3 | cytokinin dehydrogenase 7 | CKX7_ARATH |
| *LOC8258614* | 3 | 3-ketoacyl-CoA synthase 10 | KCS10_ARATH |
| *LOC8258617* | 3 | probable serine/threonine-protein kinase yakA | -- |
| *LOC8259027* | 3 | pectinesterase/pectinesterase inhibitor PPE8B | PME_PRUPE |
| *LOC8259049* | 3 | auxin-responsive protein SAUR76 | SAU76_ARATH |
| *LOC8259062* | 3 | 21 kDa protein precursor, putative | PMEI3_ARATH |
| *LOC8281151* | 3 | protein DEEPER ROOTING 1 isoform X1 | DRO1_PRUPE |
| *LOC8281160* | 3 | uncharacterized protein LOC8281160 | -- |
| *LOC8281164* | 3 | protein LURP-one-related 6 | LOR6_ARATH |
| *LOC8281165* | 3 | guanine nucleotide-binding protein subunit gamma 3 isoform X1 | GG3_ARATH |
| *novel.345* | 3 | hypothetical protein H0E87_012651 | BGA15_ARATH |

^a^ Detailed information can be search online at Uniprot (https://www.uniprot.org/uniprotkb/)
